# Phoneme discrimination and localization performance in children with cochlear implants and contralateral auditory brainstem implants with inner ear malformations

**DOI:** 10.1007/s00405-025-09474-x

**Published:** 2025-05-30

**Authors:** Erva Degirmenci Uzun, Merve Ozbal Batuk, Gonca Sennaroglu, Levent Sennaroglu

**Affiliations:** 1 Department of Audiology, Faculty of Health Science, Izmir Bakircay University, Izmir, Turkey; 2 Department of Audiology, Faculty of Health Science, Hacettepe University, Ankara, Turkey; 3 Deparment of Otolaryngology, Faculty of Medicine, Hacettepe University, Ankara, Turkey

**Keywords:** Cochlear implants, Auditory brainstem implants, Speech perception, Sound localization, Quality of life

## Abstract

**Purpose:**

This study compares unilateral and bilateral phoneme discrimination (PD) and azimuth localization abilities in children with severe inner ear malformations and cochlear nerve deficiencies who use a cochlear implant (CI) and a contralateral auditory brainstem implant (ABI).

**Methods:**

A total of 17 children between the ages of 7 and 18 years with CI and contralateral ABI were included in the study. PD and localization tests were evaluated in three conditions: CI only, ABI only, and bilateral. All subjects completed a self-report Hearing-Related Quality of Life (HRQoL) scale.

**Results:**

Statistical analysis revealed that PD scores were significantly higher in the bilateral condition than in the ABI condition alone (*p* = 0.003). However, no significant differences were found between the CI and ABI or between the CI and bilateral conditions. Similarly, no statistically significant differences in localization performance were found between the three conditions (*p* > 0.05). Regression analysis identified HRQoL as a significant predictor of bilateral PD (*p* < 0.05), while ABI localization was a significant predictor of bilateral localization (*p* < 0.05). Additionally, bilateral PD was found to be a significant predictor of HRQoL (*p* = 0.001).

**Conclusion:**

While the performance of CI alone and bilateral PD is similar, there is a significant difference between the performance of ABI alone and bilateral PD. Therefore, in cases with an ABI, bilateral stimulation should be maintained by continued use of a CI in the contralateral ear, even in the presence of severe inner ear malformation and/or cochlear nerve deficiencies. In the long term, comparable levels of PD discrimination and localization performance can be achieved by using two different modalities.

## Introduction

Historically, various classifications and definitions of inner ear malformations (IEM) have been established [1, 2]. The most widely utilized classification today, developed by Sennaroglu and Saatci [3], categorizes IEM into eight groups based on radiologic appearance: labyrinthine aplasia, including Michel deformity, rudimentary otocyst, cochlear aplasia, common cavity, incomplete partition (IP) of the cochlea, cochlear hypoplasia, large vestibular aqueduct (LVA) syndrome, and cochlear aperture (CA) anomalies.

The (re)habilitation process in IEM is more challenging than in deaf individuals with typical inner ear anatomy [4]. Although the use of hearing aids may seem unnecessary in cases of severe IEM, it may be important in some cases. It helps the child become accustomed to wearing the device and helps the family prepare for the habilitation process [5]. However, the majority of cases — with the presence of the cochlear nerve (CN) — are candidates for cochlear implants (CIs). Unlike typical inner ear anatomy, the performance outcomes of cochlear implantation in cases with IEM are highly variable and depend on factors such as the type and severity of the malformation [6]. Moreover, speech perception outcomes following cochlear implantation are likely to be lower than in children with CIs, who have a typical inner ear anatomy [7].

There are also cases with severe-to-profound SNHL who either do not benefit from CIs or are not candidates for CI due to anatomical factors such as cochlear aplasia or CN deficiency, which preclude adequate stimulation of the auditory nerve [10]. In such cases, auditory brainstem implants (ABIs) are employed, designed to bypass the auditory nerve and directly stimulate the cochlear nuclei located in the brainstem [8]. It has been observed that auditory perception abilities among children using ABI vary significantly between individuals [9]. Despite these differences, children who regularly use their ABIs can develop auditory and open-set speech perception skills to varying degrees [10].

In some cases with severe malformations, the choice between CI and ABI can be challenging [11]. In situations where insufficient progress is observed with a CI, bilateral stimulation can be achieved through sequential bilateral rehabilitation by placing an ABI on the contralateral, more malformed side [12]. According to the latest consensus statement, in cases of hypoplastic CN or cochleovestibular nerve (CVN), the combination of CI and ABI for bilateral stimulation may be a more appropriate intervention than the use of CI alone, as suggested by other studies [11]. Children with IEM require more auditory input than those with normal cochlear anatomy. Using both a CI and a contralateral ABI is crucial for children with IEM who require more auditory input. In such cases, when CN deficiency is present, the first option should be to try a CI. If the contralateral ear lacks a CN, an ABI should be provided without delay as the most effective form of stimulation [13].

The underlying reasons for the performance differences between CI and ABI remain unclear. Although both modalities use similar signal processing strategies and a comparable number of stimulating electrodes, they differ in the site of electrical stimulation, namely, the cochlea for CI and the cochlear nucleus for ABI. Two main hypotheses have been proposed to explain this discrepancy. One of them is the selectivity hypothesis, which suggests that ABIs may not establish selective contact with the tonotopic dimension of the cochlear nucleus, thereby limiting the number of independent spectral information channels. If the electrodes activate a broad neural population, this may lead to reduced spectral resolution due to poor place specificity in neural excitation [14]. The bypass hypothesis provides an alternative explanation. It suggests that the differences in speech understanding performance between the two modalities may be due to the bypassing of specialized neural circuits within the cochlear nucleus during ABI stimulation. Due to the complex structure of the cochlear nucleus, the auditory system may not support projections to higher auditory centers equally when stimulated by CI and ABI modalities [14]. However, the ability of some cases with severe IEM and CN deficiencies to develop speech understanding skills comparable to those of CI users may rule out these two hypotheses. However, when evaluating tumor and non-tumor cases, performance differences related to etiology remain largely uncertain [13–15].

Given the purpose of utilizing the aforementioned auditory modalities, the importance of auditory and speech perception outcomes is indisputable. Perceptual processes, such as recognizing and understanding auditory stimuli, are essential for auditory development in children [16]. Spatial hearing is another important auditory skill. It can be defined as “the ability to detect, locate, and identify sounds in the environment by using auditory cues from multiple directions, the perception of the spatial arrangement of sound sources” [17]. The ability to distinguish sounds according to their location plays a crucial role in auditory scene analysis and the process of separating auditory objects from background noise. In other words, spatial hearing provides cues about the number and location of environmental sources and objects, as well as the dimensions of enclosed spaces [18, 19].

The functioning of auditory-perceptual processes at different levels of the auditory system is one of the key areas of interest in audiology research. However, how the electrically stimulated auditory system processes and/or integrates this information at different levels remains poorly understood. Moreover, it is particularly intriguing to explore how these processes unfold when different modalities are employed in each ear. Data in the literature on how speech perception and sound localization skills are achieved in children with IEM using CI with contralateral ABI across different modalities are quite limited. In addition, how and to what extent CI or ABI contributes to these skills remains unclear. Existing data are mostly derived from studies conducted in adults with tumor etiologies or non-tumor case reports [20, 21]. In this study, we aimed to evaluate the audiological outcomes of children using a CI with a contralateral ABI through a comprehensive approach, to investigate how the outcomes vary between the two modalities, and to examine how each modality contributes to bilateral listening. Based on this, the present study evaluated phoneme discrimination (PD), azimuth localization, and hearing-related quality of life (HRQoL) in children using CI with contralateral ABI.

## Methods

### Ethical considerations

This study was approved by the Non-Interventional Clinical Research Ethics Board of Izmir Bakircay University, with decision number 449, dated December 17, 2021. Written informed consent was obtained from all the children and their parents.

### Subjects

A total of 17 subjects between the ages of 7 and 18 years, who had received a CI and a contralateral ABI and who were being followed up at the Departments of Otolaryngology and Audiology at Hacettepe University, were included in this study.

All subjects were children who had received bilateral stimulation for at least 12 months, were native Turkish speakers, and consistently used their speech processors for at least 10 h per day. Each subject presented with various IEMs in at least one ear and had been receiving regular auditory rehabilitation since their initial implantation. Children with additional disabilities, diagnosed neurological and/or psychiatric disorders, inability to cooperate during testing or irregular use of both speech processors were excluded from the study.

Descriptive data of all subjects regarding gender, caregiver education level, CI and ABI sides, chronological age, age at implantation, duration of bimodal use, inter-implant interval, and duration of bimodal use are presented in Table 1.

**Table 1:** Demographics

		Number (*n* = 17)
Gender, n (%)	*Female*	9 (47.1)
	*Male*	8 (52.9)
Primary caregiver education level, n (%)	*Primary and middle school*	6 (35.3)
	*High school*	6 (35.3)
	*College*	5 (29.41)
CI side, n (%)	*Right*	8 (47.1)
	*Left*	9 (52.9)
ABI side, n (%)	*Right*	9 (52.9)
	*Left*	8 (47.1)
	**Mean Value ± Standard Deviation** **(range)**	
Chronological age (mo)	105.88 ± 23.35 (84–153)	
Age at CI surgery (mo)	31.71 ± 18.02 (16–76)	
Duration of CI use (mo)	69.35 ± 34.84 (12–120)	
Age at ABI surgery (mo)	45.47 ± 16.01 (26–85)	
Duration of ABI use (mo)	64.76 ± 26.26 (14–111)	
Inter-implant interval (mo)	25.59 ± 21.32 (9–91)	
Duration of bimodal use (mo)	55.65 ± 29.38 (12–111)	

CI = cochlear implant; ABI = auditory brainstem implant; mo = months; *n* = sample size

Individual data for the subjects, including chronological age, age at implantation, processor and implant information, as well as computed tomography (CT) and magnetic resonance imaging (MRI) report details, are presented in Table 2. All participants, except for participants 5 and 6, received their CIs first, followed by ABIs in the contralateral ear. Participants 5 and 6 received their ABIs first, followed by their CIs. No case involved the initial implantation of a CI and its subsequent conversion to an ABI in the same ear (Table 2).

**Table 2:** Individual clinical characteristics

No.	Age (mo)	Age at CI surgery (mo)	Age at ABI surgery (mo)	Processor (CI)	Implant (CI)	Processor (ABI)	Implant (ABI)	CT (CI side)	MRI (CI side)	CT (ABI side)	MRI (ABI side)
1	114	17	37	Nucleus CP1000	CI422	Nucleus CP1000	ABI541	CC	CCVN	CAP + DV	CNA
2	85	29	40	Medel Sonnet	Synchrony	Medel Sonnet	Concerto	CH I	CNA	CAP	CNA
3	96	23	39	Oticon Saphyr	Digisonic SP	Oticon Saphyr	Digisonic SP	CAA	CNA	CAA	CNA
4	84	23	40	Nucleus CP1000	CI512	Medel Sonnet	Concerto	CH III	Normal	CH III	CNH
5	85	73	30	Medel Rondo 2	Sonata	Medel Opus 2	Concerto	CH I	CNA	CH III	CNA
6	88	76	26	Medel Rondo 3	Synchrony	Medel Rondo 1	Concerto	CH II	CNH	RO	CNA
7	100	21	41	Nucleus CP810	CI24RE	Nucleus CP810	ABI541	CAS	CNH	Michel	CNA
8	99	25	85	Medel Rondo 2	Sonata	Medel Rondo 2	Concerto	CAS	CNH	CAP	CNA
9	132	16	48	Medel Rondo 2	Sonata	Oticon Saphyr	Digisonic SP	CAS	CNH	CAA	CNA
10	131	36	54	Medel Opus 2	Sonata	Oticon Saphyr	Digisonic SP	CC	C CVN	Michel	CNA
11	110	16	33	Oticon Saphyr	Digisonic SP	Oticon Saphyr	Digisonic SP	IP I	Normal	IP I	CNH
12	140	23	39	Nucleus CP1000	CI512	Nucleus CP1000	ABI24M	CH I	CNA	CH I	CNA
13	153	33	42	Nucleus CP910	CI512	Nucleus CP910	ABI24M	Normal	Normal	IP I	CNA
14	131	28	40	Oticon Saphyr	Digisonic SP	Oticon Saphyr	Digisonic SP Evo	CAS	CNA	CAS	CNA
15	87	28	59	Medel Opus 2	Sonata	Oticon Saphyr	Digisonic SP	CH I	CNH	CH III	CNA
16	80	23	40	Nucleus CP910	CI512	Nucleus CP910	ABI24M	CH II	CNH	CAP	CNA
17	85	49	80	Medel Opus 2	Pulsar	Medel Opus 2	Concerto	CC	CCVN	CC	C CVN

ABI = auditory brainstem implant; CAA = cochlear aperture aplasia; CAP = cochlear aplasia; CAS = cochlear aperture stenosis; CC = common cavity; CCVN = common cochleovestibular nerve; CH I = cochlear hypoplasia type I; CH II = cochlear hypoplasia type II; CH III = cochlear hypoplasia type III; CI = cochlear implant; CNA = cochlear nerve aplasia; CNH = cochlear nerve hypoplasia; CT = computed tomography; DV = dilated vestibule; IP-I = incomplete partition type I; mo = months; MRI = magnetic resonance imaging; RO = rudimentary otocyst

### Materials and procedure

After collecting the demographic data from all subjects who met the inclusion criteria, the PD and localization tests were administered, and the HRQoL evaluation was applied. All children completed both the PD and localization tests in a double-walled soundproof booth provided by the Industrial Acoustics Company. Stimuli and speaker calibrations were performed using a Wintact WT1357 sound level meter before the assessments. All assessments took approximately three hours to complete. All children completed PD and localization tests under three listening conditions: (1) CI only, (2) ABI only, and (3) CI and ABI together. The assessments were conducted on all children in a randomized order. In all cases, all tasks were completed within the same day, with the number of sessions scheduled according to the child’s level of cooperation and needs.

### Phoneme discrimination task

#### Test equipment and setup

All subjects underwent the PD test, which is part of the Auditory Speech Sound Evaluation Test (A§E) psychoacoustic test battery [22]. The test is included within the Otoconsult software, and both installation and use were performed according to the manufacturer’s instructions [23]. The software package was delivered via a sound card and connected speakers. Before each test, the system was calibrated to 70 dB SPL using the calibration mode available in the software and a sound level meter. Each subject was seated in a chair, equidistant (1 m) from the two loudspeakers. The loudspeakers were positioned at + 90° and − 90° azimuth. In the unilateral (CI-only or ABI-only) test condition, the stimuli were presented through the speaker on the side of the evaluated implant.

#### Test stimuli and procedure

It is an oddity test in which two speech sounds are presented and a listener is conditioned to react to an odd speech sound. In other words, the test is fundamentally based on the behavioral response —specifically, raising a hand for this study—to identifying a target stimulus, which is presented once among a series of repetitive background stimuli [22]. The PD task consists of a list of 20 phoneme pairs, which are as follows: /a/-/r/; /u/-/i/; /z/-/s/; /ɘ/-/u/; /o/-/a/; /ɘ/-/a/; /v/-/z/; /ɘ/-/i/; /ɛ/-/a/; /u/-/ʃ/; /ɘ/-/ɛ/; /m/-/z/; /u/-/y/; /u/-/o/; /y/-/i/; /u/-/a/; /s/-/ʃ/; /i/- /ɛ/; /i/-/a/; /ɘ/-/o/. In each phoneme pair, the first phoneme was presented as a background stimulus, while the second function served as the target stimulus. The number of background sounds varied randomly between three and eight. The target stimulus was presented only once, and the subjects were expected to identify the target stimulus among the repeated background sounds. The duration of both the background and target phonemes was 625 ms. The background phoneme was repeated at intervals of 850 ms, and both the background and target phonemes were presented through the same loudspeaker. The discrimination task does not require language proficiency and enables the assessment of a child’s ability to distinguish between different phonemes [22].

The subjects were introduced to the task in a training mode, where they were instructed to raise their hands when they heard the odd target stimulus before beginning the test. The training mode was designed to ensure the subjects’ cooperation and adaptation to the test. Once the subject was ready, the test mode commenced [22]. After completing the test under three different conditions, the Audioqueen software provided a PD score as a percentage (%) whenever the “correct” or “incorrect” option was selected for the listener’s response.

### Sound localization

#### Test equipment and setup

The sound localization abilities of all subjects were assessed using the Azimuth Localization Test, which is part of the A§E psychoacoustic test battery. This test is integrated into the Otoconsult software, and the evaluations were conducted according to the manufacturer’s instructions for installation and use [24].

The test utilized SoundC, which requires hardware that includes seven speakers positioned horizontally between − 60 and 60 degrees, an amplifier system, and an 8-channel sound card to manage the speakers. Seven speakers numbered from − 3 (left) to 3 (right) were positioned in a semicircular plane with 20-degree intervals. The SoundC setup was calibrated by adjusting all speakers to the same level, approximately at the maximum volume. During the calibration phase, the calibration mode within the localization test software was selected, allowing for the activation of one or more channels by choosing from the right, left, or both, and labels were assigned to each channel. The calibration signal was then played through the software while the sound level was measured with a sound level meter. Necessary adjustments were made to ensure a reading of 70 dB SPL on the sound level meter [24, 25].

#### Test stimuli and procedure

A narrow-band noise with a center frequency of 4000 Hz was used as a stimulus and presented at 70 dB SPL to evaluate sound localization. Each stimulus has a duration of 3 s and is presented five times. The test software randomly selected a speaker for each stimulus, with a total of 35 test stimuli automatically presented by the system. The subjects were seated directly in front of the speaker at 0° azimuth (speaker number 0), ensuring that their heads were aligned with the speaker, and they were instructed to identify the source of the sound without moving their heads. The response mode allowed the subjects to either point to the speaker with their fingers or verbalize the shapes or numbers placed on the speakers. The subjects were asked to identify the speaker from which the sound originated, and their responses were recorded by selecting the corresponding speaker in the computer system [25, 26] The children went through a training mode provided in the software to ensure their cooperation and adaptation to the test, before the start.

After all stimuli were presented and the subjects’ responses were recorded in the system, the software generated an XY graph displaying the median responses as a function of the actual sound sources. Sound localization performance was presented as median values and the root mean square (RMS) error, both calculated automatically by the software. A lower RMS error indicates better localization ability, while a higher error indicates worse sound localization performance. Negative and positive values represent directionality based on loudspeaker placement, specifically, left and right, respectively.

### Hearing-related quality of life

The Hearing Environments and Reflection on Quality of Life (HEAR-QL) scale was designed by Streufert [27] for children and adolescents in 2008 and shown to be valid and reliable by Umansky et al. (2011) [28] and Rachakonda et al. (2014) [29]. In the present study, the Turkish version, adapted by Budak et al. (2023), was used [30]. The scale has two versions: the HEAR-QL-26 for children and the HEAR-QL-28 for adolescents. The HEAR-QL-26, consisting of 26 items, was used for subjects aged 7–12 years. This version of the scale evaluates deaf children on three subscales: environment, activities, and emotions. The adolescent version, HEAR-QL-28, consisting of 28 items, was used for subjects aged 13–18 years. This version assesses four subcategories: hearing conditions, social interactions, school challenges, and emotions.

Each item on the questionnaire utilized a Likert-type scale, with scores ranging from 0 to 100. The children were instructed to indicate how often each item had been a problem for them in the past month by selecting from the following response options: “never” (1), “almost never” (2), “sometimes” (3), “often” (4), or “always.” Responses were converted into corresponding scores as follows: never = 100, almost never = 75, sometimes = 50, often = 25, and almost always = 0. Both overall mean scores and subgroup scores were calculated, with higher scores reflecting better perceived HRQoL [30].

### Statistical analysis

The data obtained from this study were analyzed using SPSS version 24 (IBM Corp., Somers, NY, USA). The Kolmogorov–Smirnov and Shapiro–Wilk tests and visual methods (histograms and probability plots) were employed to assess whether the variables followed a normal distribution. Descriptive statistics for all subject characteristics (e.g., gender, chronological age, caregiver education level, duration of bilateral use, and age at implantation) were examined.

For multiple comparisons, the Friedman test was used to compare the numerical variables between the three listening conditions—CI, ABI, and bilateral—regarding PD and sound localization values, as the assumption of normality was not met. The test accounts for the fact that the data are paired. Post hoc analysis was performed using the Wilcoxon signed-rank test with Bonferroni correction. Comparisons were made between the CI and ABI, CI and bilateral, and ABI and bilateral conditions. Spearman’s correlation coefficient (*r*) was utilized to examine the relationships between age at implantation, duration of CI or ABI use, duration of bilateral use, inter-implant intervals, and test results. Linear regression analysis was used to evaluate the factors affecting test results. The model’s goodness of fit was evaluated using the R-squared value. A p-value of less than 0.05 was considered statistically significant.

## Results

### Phoneme discrimination outcomes

**Fig. 1:**
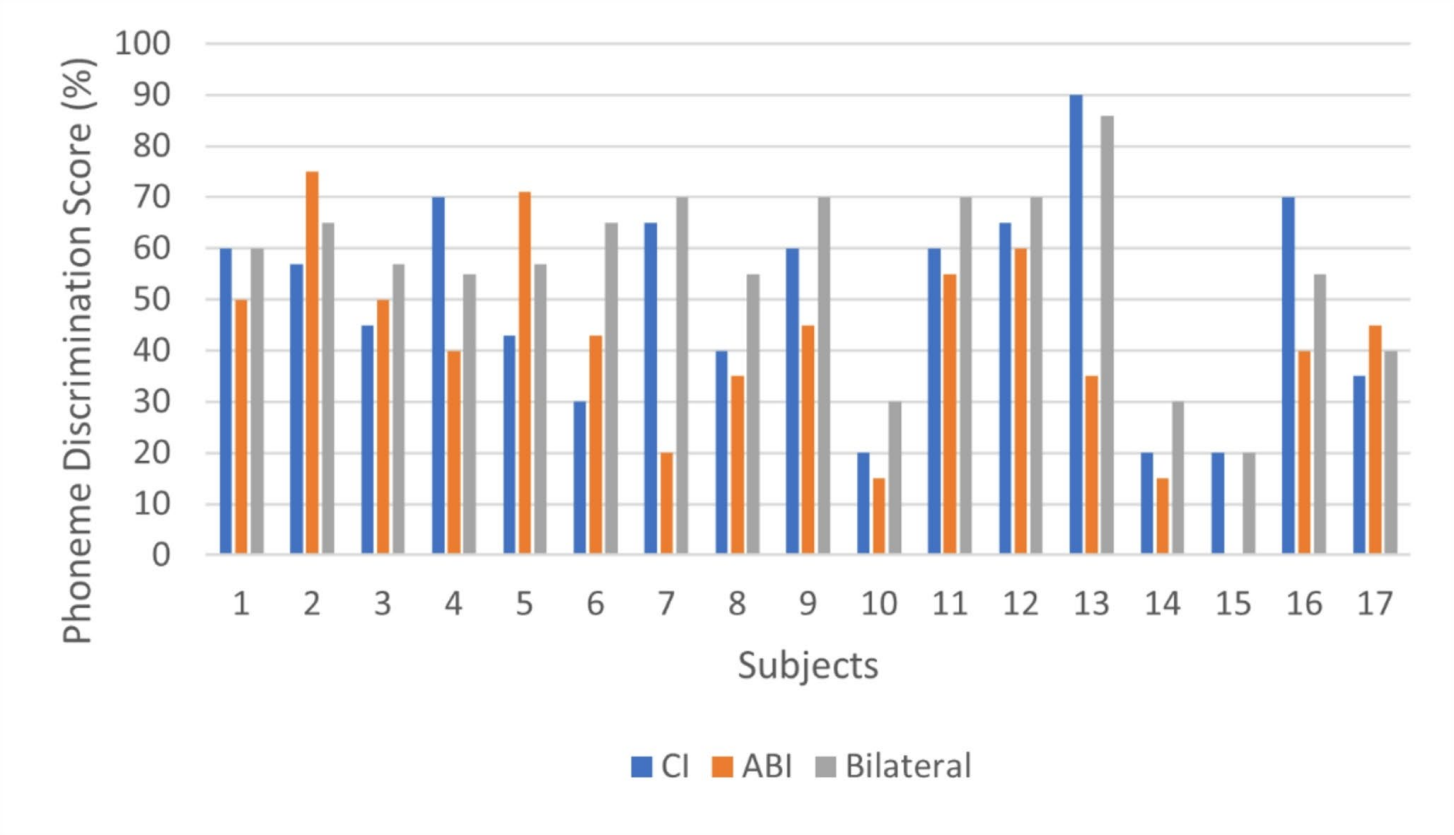
PD test scores of the subjects: ABI = auditory brainstem implant; CI = cochlear implant

**Fig. 2:**
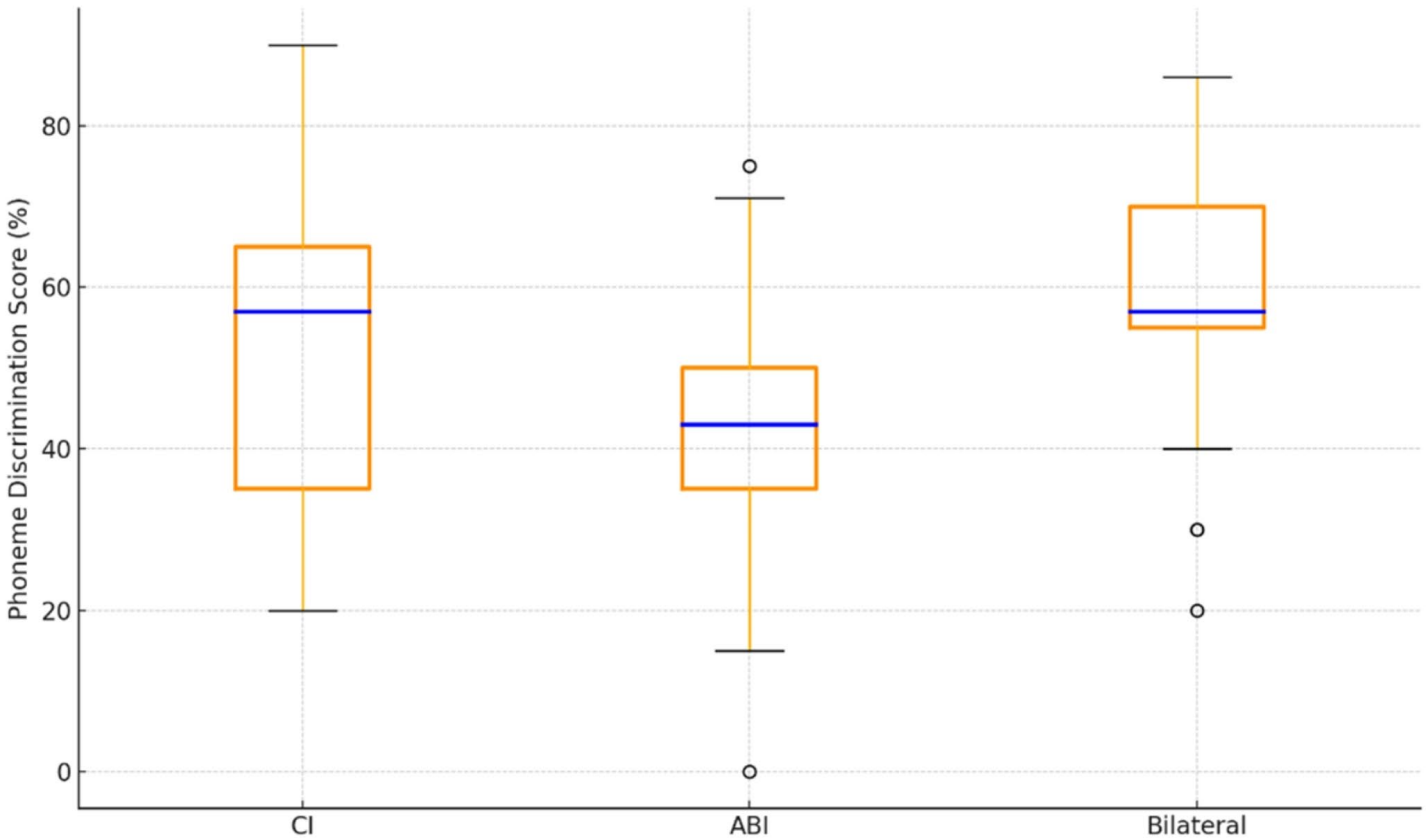
PD score box plots: ABI = auditory brainstem implant; CI = cochlear implant

Individual PD test results for each subject, as well as comparative box plots of the PD test across different test conditions, are shown in Figs. 1 and 2, respectively.

The median PD score for the CI-only condition was 57%, with 20% and 90% minimum and maximum scores, respectively. For the ABI condition, the median PD score was 43%, with scores ranging from 0 to 75%. For the bilateral condition, the median PD score was 57%, and the minimum and maximum scores were 20% and 86%, respectively. Statistical analysis revealed a significant difference between the three listening conditions (χ²(2) = 11.030, *p* = 0.004) with a medium effect size (Kendall’s W = 0.324).

Post hoc analysis showed that the bilateral PD score was statistically significantly higher than the ABI PD score (Z = -3.112, *p* = 0.003). No significant differences were observed between the ABI and CI alone (Z = -1.547, *p* > 0.05) or between the CI alone and bilateral PD scores (Z = -1.881, *p* > 0.05). 12 of the 17 participants demonstrated better PD performance with the CI compared to the ABI. The remaining 5 participants performed better with the ABI compared to the CI-only condition.

### Sound localization outcomes

**Fig. 3:**
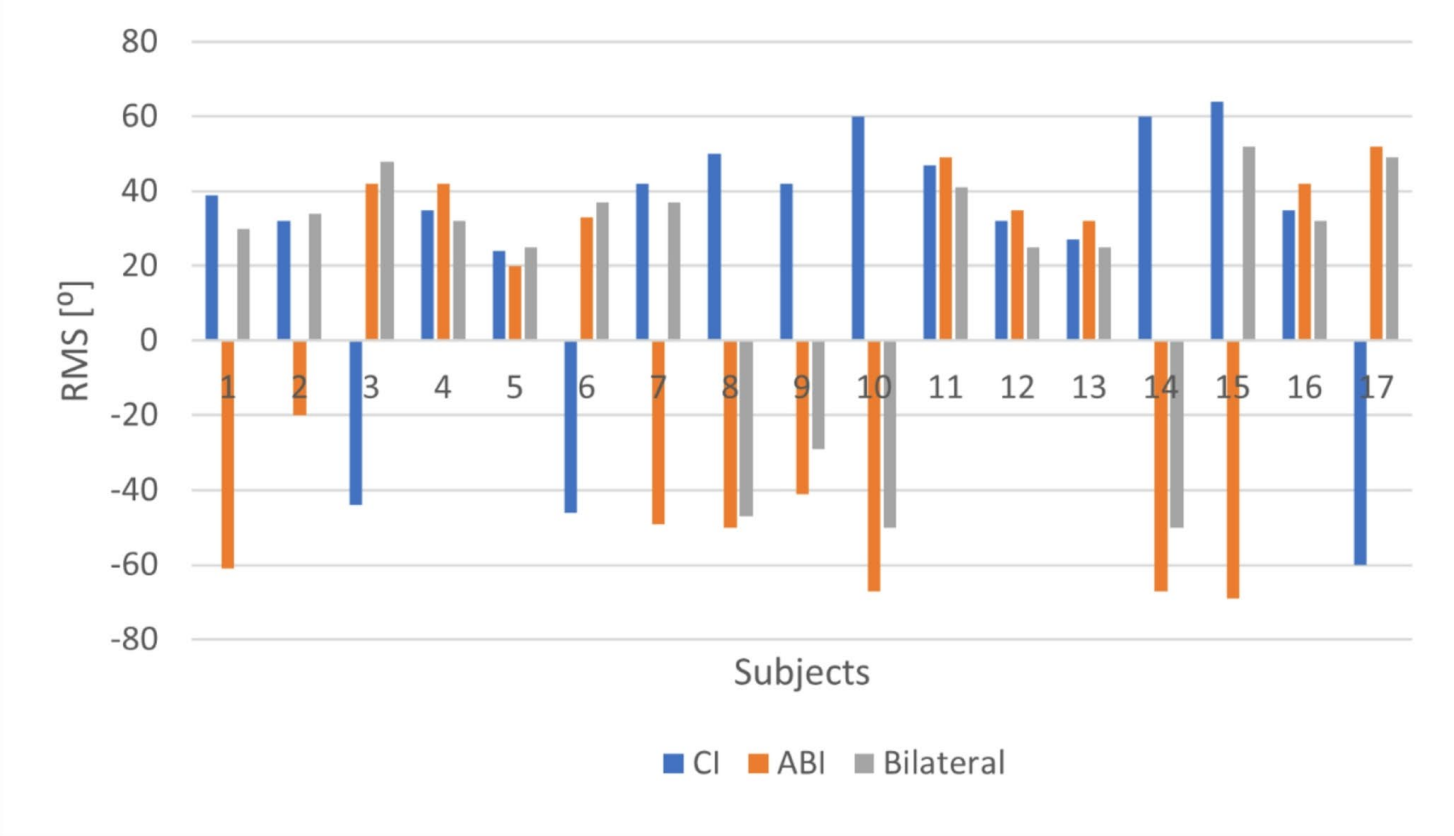
Localization test results of the subjects: ABI = auditory brainstem implant; CI = cochlear implant; RMS = root mean square

**Fig. 4:**
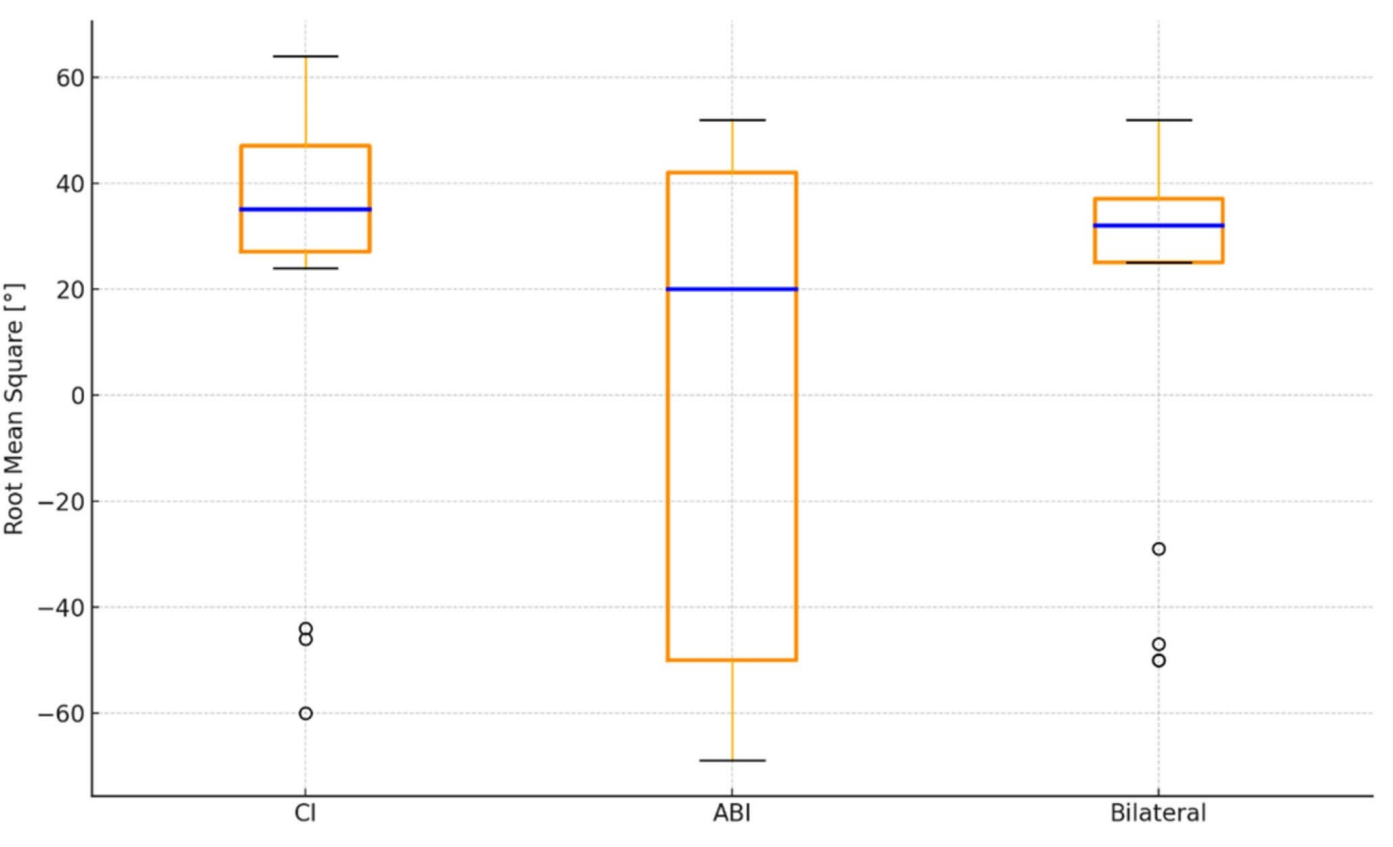
Localization results box plots: ABI = auditory brainstem implant; CI = cochlear implant; RMS = root mean square

The azimuth localization test results for each subject, along with comparative box plots under three different test conditions, are shown in Figs. 3 and 4, respectively. Localization occurred on the CI side for eight subjects, while it occurred on the ABI side for nine subjects.

The median localization value for the CI-only condition was 35 RMS errors, with − 60 to 64 RMS errors for the minimum and maximum values, respectively. For the ABI-only condition, the median localization was 20, with a range of -69 to 52. For the bilateral condition, the median localization value was 32, with a range of -50 to 52 RMS errors. No statistically significant difference was found between the RMS values compared across the three conditions—CI only, ABI only, and bilateral—(χ²(2) = 1.529, *p* > 0.05), with a very weak effect size (Kendall’s W = 0.045). Out of 17 participants, 13 demonstrated better sound localization performance under the bilateral listening condition. The remaining 4 participants performed better with the ABI compared to the CI-only and bilateral conditions.

### Hearing-related quality of life

**Fig. 5:**
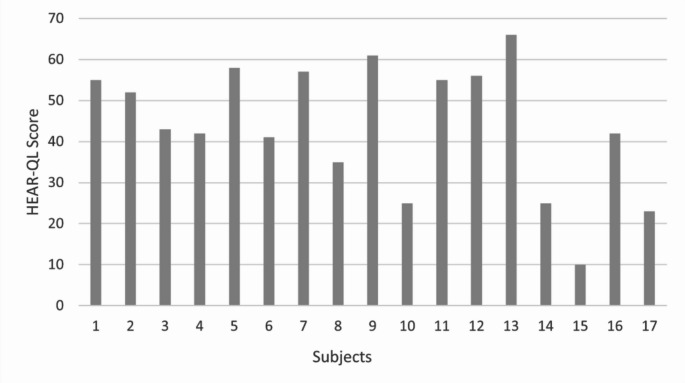
Subjects’ hearing-related quality of life scale scores

The median HEAR-QL scale score for the subjects was 43, with a range of 10 to 66. The individual scale scores are illustrated in Fig. 5.

### Correlations

Correlations between all the variables were checked for co-linearity prior to the linear regression analysis. The relationship between the test results is shown in Table 3. According to the analysis, a moderately positive correlation was found between the ABI PD score and the ABI localization. No statistically significant correlations were observed between the CI PD score and CI localization, or between the bilateral PD score and bilateral localization. A strong positive correlation was found between the bilateral PD score and HRQoL; however, no statistically significant correlation was observed between bilateral localization and HRQoL.

**Table 3:** Correlations between test results

	CI Localization		ABI Localization		Bilateral PD		Bilateral Localization	
	*r*	*p*	*r*	*p*	*r*	*p*	*r*	*p*
CI PD	-0.253	0.326	•	•	•	•	•	•
ABI PD	•	•	0.525	0.030*	•	•	•	•
Bilateral PD	•	•	•	•	•	•	0.009	0.974
HRQoL	•	•	•	•	0.873	0.001*	-0.192	0.460

ABI = auditory brainstem implant; CI = cochlear implant; HRQoL = hearing-related quality of life;

*p* = significance level; *r* = correlation coefficient

*Significant: *p* < 0.05

In addition, the correlations between chronological age, age at implantation, inter-implant intervals, and duration of implant use with the test results were also investigated. The correlation results are presented in Table 4.

**Table 4:** Correlations between test results and demographics

	Chronological age		Age at CI surgery		Age at ABI surgery		Duration of CI use		Duration of ABI use		Duration of bimodal use		Inter-implant interval	
	*r*	*p*	*r*	*p*	*r*	*p*	*r*	*p*	*r*	*p*	*r*	*p*	*r*	*p*
CI PD	•	•	-0.516	0.034*	•	•	0.370	0.144	•	•	•	•	•	•
CI Localization	•	•	-0.376	0.137	•	•	0.325	0.204	•	•	•	•	•	•
ABI PD	•	•	•	•	-0.598	0.011*	•	•	0.070	0.789	•	•	•	•
ABI Localization	•	•	•	•	-0.321	0.209	•	•	0.125	0.633	•	•	•	•
Bimodal PD	0.437	0.079	•	•	•	•	•	•	•	•	0.456	0.066	-0.263	0.307
Bilateral Localization	-0.510	0.037*	•	•	•	•	•	•	•	•	-0.328	0.202	0.117	0.656
HRQoL	0.381	0.132	-0.372	0.141	-0.371	0.142	•	•	•	•	•	•	-0.303	0.237

ABI = auditory brainstem implant; CI = cochlear implant; HRQoL = hearing-related quality of life; PD = phoneme discrimination; *p* = significance level;

*r* = correlation coefficient

*Significant: *p* < 0.05

A moderate negative correlation was observed between chronological age and bilateral localization, while no significant correlation was found between chronological age and either the bilateral PD score or HRQoL. A moderate negative correlation was observed between CI age and CI PD score, as well as between ABI age and ABI PD score. However, no statistically significant correlations were found between CI age and CI localization or between ABI age and ABI localization. Similarly, no correlations were detected among CI age, ABI age, and HRQoL. Additionally, no significant relationships were observed between implant usage durations and test results, nor was there a correlation between the inter-implant interval and bilateral PD, bilateral localization, or HRQoL.

### Predictors of phoneme discrimination, sound localization, and hearing-related quality of life

Table 5 shows the results of the linear regression analyses. To determine the factors affecting the bilateral PD score, a regression analysis model was carried out using ABI PD, ABI localization, CI PD, CI localization, HRQoL, and the duration of bilateral use variables. The model identified HRQoL as a significant predictor of bilateral PD (*p* < 0.05). The model’s explanatory power was determined to be 86.5%, with a 1.015-unit increase in bilateral PD for each 1-unit increase in HRQoL.

**Table 5:** Linear regression analysis results

Predictors				%95				
	*B*	*ß*	*t*	Lower	Upper	*R* ^*2*^	*F*	*p*
*Bilateral Phoneme Discrimination*								
HRQoL	1.015	0.930	9.79	0.794	1.236	0.865	96.003	< 0.001*
* Intercept*	11.641		2.418	1.378	21.905			0.029
*Bilateral Localization*								
ABI Localization	0.426	0.577	2.737	0.094	0.759	0.333	7.490	0.015*
* Intercept*	19.049		2.418	2.566	34.871			0.021
*Hearing-Related Quality of Life*								
Bilateral PD	0.852	0.930	9.798	0.667	1.038	0.865	96.003	0.001*
* Intercept*	-3.991		-0.783	-14.858	6.877			0.446

ABI = auditory brainstem implant; B: non-standardized regression coefficient; *β* = standardized regression

coefficient; CI = cochlear implant; *F* and *t*: F and t statistic; HRQoL = hearing-related quality of life;

*p* = significance level; PD = phoneme discrimination; *R*^*2*^ *=* coefficient of determination

Regression analyses were carried out using bilateral PD, ABI PD, ABI localization, CI PD, CI localization, HRQoL, and duration of bilateral use as dependent variables to examine factors affecting the bilateral localization value (RMS). The regression model identified ABI localization as a significant predictor of bilateral localization (*p* < 0.05). The model’s explanatory power was determined to be 33.3%, with a 0.426-unit increase in bilateral localization for each 1-unit increase in ABI localization.

Finally, to examine factors affecting HRQoL value, bilateral localization, bilateral PD, ABI PD, ABI localization, CI PD, CI localization, CI age, and caregiver education level were included in the regression analysis model. The regression model identified bilateral PD as a significant predictor of HRQoL (*p* = 0.001). The model’s explanatory power was determined to be 86.5%, with a 0.852-unit increase in HRQoL for each 1-unit increase in bilateral PD.

## Discussion

The present study highlights PD, sound localization performance outcomes, and HRQoL in children using a CI with a contralateral ABI. It also examines the contributions of CI and ABI to PD and localization skills, as well as the variables that affect these skills. To the best of our knowledge, this study presents the first reported comprehensive outcomes of the largest sample of children using a CI with a contralateral ABI.

### Phoneme discrimination

There was notable variability in PD performance, especially between children with CN anomalies among children with CIs and contralateral ABIs, particularly those with CN anomalies. While children with normal CN achieved CI-only PD scores ranging from 60 to 90%, those with CN anomalies demonstrated lower scores ranging from 20 to 70%. In this study, the ear that showed better behavioral responses during the preoperative evaluation received the CI. The ABI was preferred for the ear with poorer anatomical and audiological characteristics. Although ABI is a proven and effective auditory implant method, the exact nature of the region it stimulates remains unclear. Its effectiveness is still more limited compared to CI. Therefore, in ears with CN deficiency where CI electrode placement is possible and at least minimal auditory response is present, CI has consistently been our first choice. Although statistically comparing CI users with and without CN anomalies is challenging due to sample size limitations, the observed differences in performance between those with and without CN anomalies are generally consistent with the findings reported in the literature [31, 32]. The variability in outcomes requires an understanding of the factors that influence these results. Anatomical abnormalities such as anastomotic connections between the cochlear and vestibular nerves or undetectable cochlear fibers due to MRI limitations may hinder effective stimulation [33, 34].

While ABI offers a rehabilitation option for children with severe IEM and CN anomalies who are not candidates for CI, outcomes are often less predictable and generally inferior compared to CI. In this study, ABI-only PD scores ranged from 0 to 75%, with lower scores linked to older age at implantation. Early intervention, particularly before three years of age, is critical to maximize outcomes [35, 36]. However, ABI outcomes often require longer rehabilitation periods because auditory skill development with ABI is slower than with CI [9].

The combination of CI and contralateral ABI holds the potential to enhance auditory outcomes, particularly in children with severe auditory pathologies. Friedmann et al. (2018) demonstrated the effectiveness of this approach in four children, showing significant improvements in open-set speech perception with bilateral stimulation [35]. In the present study, bilateral stimulation consistently yielded higher PD scores than ABI alone, with PD performance ranging from 20 to 86%. However, bilateral performance was not statistically superior to CI alone, indicating that further exploration of bilateral dynamics is needed. Individual-level analysis revealed that nine participants achieved better outcomes with bilateral stimulation, underscoring its potential to overcome the limitations of ABI. Nevertheless, five participants showed reduced bilateral performance compared to their best unilateral outcomes. Of these, three performed better with CI alone, including two with normal CN. This suggests that interference between CI and ABI stimulation may impact outcomes, especially in cases with intact CN functionality. However, experimental and physiological studies are required to clarify these interactions.

Conversely, two participants with severe IEM on the CI side performed better with ABI alone than with bilateral conditions. These findings highlight the importance of anatomical considerations when evaluating the effectiveness of bilateral stimulation. CN anomalies, prevalent among the study cohort, likely contributed to the observed variability. Additionally, the electrical interference between the CI and ABI may further complicate bilateral integration [20]. This context raises an intriguing question about how the information transmitted from a normal CN to the brainstem interacts with the electrical stimulation provided by the ABI at the brainstem level. However, further studies are needed to elucidate the underlying mechanisms.

Given the variability in outcomes, personalized approaches remain essential when managing children with CI and ABI. Early intervention, consistent device use, and strong family support are essential to optimize auditory performance and QoL [37]. Bilateral stimulation, despite its challenges, demonstrates significant potential to improve outcomes in complex cases. However, further research is needed to refine our understanding of the mechanisms underlying bilateral interactions and interference.

### Sound localization

A review of the literature reveals a lack of studies specifically addressing sound localization skills in children with ABI or receiving CI with contralateral ABI stimulation. The current body of literature consists largely of adult case studies [38]. Research on children with congenital bilateral profound SNHL receiving bilateral CI provides insights into auditory plasticity. Studies of bilateral CI show that sound localization abilities improve with time and experience, as children develop spatial hearing strategies that align with the acoustic field [39]. However, bilateral CI and CI-ABI setups are fundamentally different, making direct comparisons problematic.

Sound localization improvements are influenced by factors such as the age at implantation, neural reserve, and auditory experience [40, 41]. In this study, no significant relationships were found between localization performance and the age at CI or ABI implantation. Factors such as cognitive and maturational development likely play a role, as binaural capacities typically mature in children by five years of age [42, 43]. All participants in this study were at least seven years old, with 12 to 110 months of bilateral auditory experience.

Sinnathuray et al. (2012) [20] presented speech perception, sound localization, and QoL outcomes in two postlingually deafened adult users of CI and contralateral ABI. In the first case, CI resulted in better sound localization than ABI, although ABI performed better when the stimulus was presented specifically to it. With bilateral stimulation, performance again favored the CI. The second case, unlike the first, demonstrated superior ABI performance, with 100% accuracy when stimuli were presented under the ABI-activated-alone listening condition. Such variability underscores the influence of individual neural reserves and device-specific interactions on sound localization outcomes [20]. Consistent with this, our study found that 13 out of 17 children showed better localization with bilateral stimulation, although this difference was not statistically significant. In four cases, localization performance was better with ABI alone, likely due to anatomical asymmetries or mismatches in neural integrity between the ears. These cases may reflect interference between CI and ABI input, as previously proposed by Sinnathuray et al. (2012) [20]. Conversely, cases with better sound localization under CI alone generally had normal CN or milder malformations. This contrast further emphasizes how outcomes are shaped by individual auditory profiles. Rather than explicitly reiterating the interference, we interpret such patterns as reflecting complex interactions between device-specific input and underlying neural substrates.

Of the 17 cases, 10 performed better with CI than with ABI, consistent with their superior PD performance under CI conditions. These cases had more CI experience and often normal cochlear or CN anatomy. In contrast, six participants showed better ABI localization due to severe malformations on the CI side. One case exhibited equal localization performance with both modalities, highlighting the variability within this heterogeneous population. Sound localization abilities rely on access to interaural time differences (ITDs), interaural level differences (ILDs), and spectral cues. While CI users may partially benefit from ILDs and ITDs depending on device synchronization, ABI users typically receive less precise spatial cues due to non-tonotopic stimulation and bypass of peripheral auditory processing. These differences may help explain the variability in localization outcomes observed in this population. In contrast, ABI users typically receive less precise spatial information, as the stimulation bypasses the auditory nerve and tonotopic organization is less specific. Consequently, ABI users often experience compromised binaural integration and fine-grained localization cues. This difference may partially explain the variability and limitations observed in localization performance among CI-ABI users [14, 15].

As noted above, the limited literature on CI-ABI users underscores the need for further investigation. Existing measurement tools, designed for typical inner ear and CN anatomy, may lack the sensitivity needed for this population. Tailored tools are needed to accurately assess sound localization in children with severe IEM or CN anomalies. Moreover, longitudinal studies and experimental research are essential to improve our understanding of the factors that influence CI-ABI localization performance. Given this complexity, existing measurement tools — often designed for typical cochlear and nerve anatomy — may not fully capture the localization abilities of CI-ABI users. Tailored assessments and longitudinal research are needed to improve our understanding of how spatial hearing develops under these unique listening conditions.

### Hearing-related quality of life

Hearing loss has a significant impact on psychosocial aspects of individuals’ lives, affecting communication, self-esteem, and social relationships. While speech perception and auditory outcomes are critical components of CI and auditory ABI evaluations, they provide only a partial view of the overall benefits [44]. To capture the broader impact of auditory interventions, QoL assessments are essential, as they reflect dimensions beyond what traditional audiological tests can measure [35].

The literature on HRQoL in children using ABI is limited. A study by Fernandes et al. (2017) [45], that evaluated the quality of life and satisfaction of 19 ABI users indicated, that parents of children using ABI were generally dissatisfied with the acoustic and psychological benefits, speech understanding performance, and aesthetics of the device. However, they observed positive effects on self-esteem, social relationships, school performance, and family dynamics. Overall, the literature indicates that the QoL outcomes for ABI users are not as favorable as those for CI users. Although some case reports suggest an improvement in QoL scores after ABI compared to preoperative levels [21], more systematic research is needed.

The combination of CI and contralateral ABI has received minimal attention in HRQoL research. Sinnathuray et al. (2012) [20] reported that two adult CI-ABI users preferred bilateral listening, noting improved satisfaction and communication in complex environments. In the present study, self-reports of children using CI with contralateral ABI showed a strong correlation between HRQoL and bilateral PD performance. This suggests that bilateral stimulation has a positive impact on HRQoL, even if it is not directly reflected in test results. However, no significant correlation was found between sound localization ability and HRQoL. While sound localization plays a crucial role in daily life, the absence of this relationship may indicate limitations in current HRQoL scales, that do not specifically target localization-related challenges.

### Strengths and limitations

The center where the study was conducted is recognized as the clinic with the largest case group worldwide. All subjects in our study represent the best-performing cases within our CI and contralateral ABI follow-up group. To control the variables in our research and standardize all conditions, many cases were excluded from the study. However, there are several limitations to this study. One of these is the inherent heterogeneity of the pathology studied, which is due to the nature of the research. However, this heterogeneity should have been minimized by ensuring that all subjects underwent audiological follow-up, radiological examinations, and surgery performed by the same team. Moreover, the current study represents the most homogeneous sample of this specific group.

Another limitation is that due to the limited sample size, variables such as the number of active electrodes and programming parameters, that could influence the outcomes, were not examined. Additionally, there were differences in the brands and models of the CI and ABI systems used by the subjects, which could potentially impact performance. However, as the subjects were experienced users, it was considered more appropriate to evaluate them in their current settings. Finally, the free-field hearing thresholds for CI and ABI were not within the speech banana of the audiogram for some subjects, particularly due to the limitations of the ABI, as discussed in the study.

Since the subjects were exposed to limited auditory input, they exhibited suprathreshold behavioral responses to pure tones. However, their SAT/SDT results using Ling’s six sounds were within the speech banana range. Additionally, subjects demonstrated substantial heterogeneity in their hearing thresholds. For these reasons, rather than relying solely on hearing thresholds, we prioritized ensuring that Ling sounds fell within the speech area. The population’s nature makes controlling every variable difficult, and accounting for all of them is not entirely feasible. In addition, the currently available hearing and language assessments were largely developed and standardized for children with typical anatomy. As a result, these tests have several limitations when applied to the population in this study, which introduces additional difficulties in evaluating all of the relevant variables. Despite these potential limitations mentioned, the present study is the first of its kind to include such a large sample size within the most challenging group to achieve developmental progress in otology and audiology follow-up.

## Conclusion

To the best of our knowledge, the present study is the first in the literature to examine and integrate PD, sound localization, and HRQoL in children using CI with contralateral ABI.

In this study, PD performance was found to be higher with bilateral stimulation than with ABI alone. Additionally, PD performance with bilateral listening and with CI alone were found to be similar. This indicates that CI users with various IEM and CN anomalies are able to perceive and discriminate phonemic-level contrasts to a significant extent. Finally, we found that the PD performance with CI and contralateral ABI in children was statistically similar. This means that in some cases where a CI with contralateral ABI is recommended for a child, similar phonemic-level performance may be seen with both devices. However, further studies with larger sample sizes are needed to generalize this finding.

Regarding long-term sound localization abilities, localization performance was similar with CI, ABI, and bilateral conditions. Significant gaps remain in the literature regarding how sound localization ability is achieved with ABI. Future studies should include experimental research designs and electrophysiological assessments to clarify these mechanisms. Additionally, longitudinal research designs are needed in this area. Finally, a significant positive correlation was observed between ABI PD performance and ABI localization performance, suggesting that children who achieve better phonemic discrimination with ABI may also demonstrate better spatial hearing performance.

## Data Availability

The data that support the findings of this study are available on request from the corresponding author.
